# Gynaecological cancer in Caribbean women: data from the French population-based cancer registries of Martinique, Guadeloupe and French Guiana (2007–2014)

**DOI:** 10.1186/s12885-020-07128-1

**Published:** 2020-07-10

**Authors:** Clarisse Joachim, Jacqueline Véronique-Baudin, Laure Desroziers, Édouard Chatignoux, Sophie Belliardo, Juliette Plenet, Jonathan Macni, Stephen Ulric-Gervaise, Jessica Peruvien, Bernard Bhakkan-Mambir, Jacqueline Deloumeaux

**Affiliations:** 1 CHU de Martinique, Pôle de Cancérologie Hématologie Urologie Pathologie, UF 1441 Registre Général des cancers de la Martinique, F-97200 Martinique, France; 2 French Network of Cancer Registries, F-31000 Toulouse, France; 3 Registre général des cancers de la Guyane, Guyane, France; 4 French National Public Health Agency, 12 rue du Val d’Osne, 94410 Saint Maurice, France; 5 Registre Général des Cancers de Guadeloupe, Centre Hospitalier Universitaire de Guadeloupe, Guadeloupe F.W. I. Route de Chauvel, 97159 Pointe-à-Pitre Cedex, France

**Keywords:** Cancer registry, Incidence, Caribbean, Women

## Abstract

**Background:**

For the first time, we present regional-level cancer incidence and world-standardized mortality rates for cancers for Martinique, Guadeloupe and French Guiana.

**Methods:**

For Martinique, Guadeloupe and French Guiana, incidence data come from population-based cancer registries, and cover the periods 2007–2014, 2008–2014 and 2010–2014 respectively. Standardized incidence and mortality rates were calculated using the world population.

**Results:**

In the 3 regions, all cancers combined represent 3567 new cases per year, of which 39.8% occur in women, and 1517 deaths per year (43.4% in women). Guadeloupe and Martinique present similar world-standardized incidence rates. Among gynaecological cancers, breast cancer, the second most common cancer type in the 3 regions, has an incidence rate 35 to 46% lower than in mainland France. On the other hand, cervical cancer has a higher incidence rate, particularly in French Guiana. For both endometrial cancer and ovarian cancer, no significant differences in incidence rates are found compared to mainland France.

Regarding mortality, world-standardized mortality rates are similar between Guadeloupe and Martinique, and higher than in French Guiana. This situation compares favourably with mainland France (all cancers). Among gynaecological cancers, the mortality rate is lower for breast cancer in all regions compared to mainland France, and also lower for ovarian cancer in Martinique and Guadeloupe, but higher (albeit non-significantly) in French Guiana.

**Conclusion:**

The ethno-geographic and socio-demographic characteristics in this population of mainly Afro-Caribbean origin could partially explain these disparities. Major disparities exist for certain cancer sites: excess incidence and excess mortality for cervical cancer; lower, but increasing incidence of breast cancer.

## Background

Cancer registries have been created progressively since the 1970s. In mainland France, registries cover approximately 16–20% of the population [1]. In the French overseas territories, population-based cancer registries (PBCRs) exist in Martinique (1981), Reunion Island (1988), French Guiana (2005) and Guadeloupe (2008). Martinique had 383,910 inhabitants as of 1 January 2014 [2]. In these territories, the exhaustiveness of coverage is > 95% in Guadeloupe and Martinique, but lower in French Guiana, due to the geographical distribution of the population in this territory. Indeed, 90% of the population is concentrated along the coast, and 99% of the territory is covered by the Amazon rain forest [3].

The PBCRs have an exhaustive data collection circuit including laboratory results, discharge reports, pathology findings, clinical patient files. Methods of cancer registration include both active and passive collection of data, using electronic and paper-based sources. The population of all three regions is covered by French national health insurance, which can be complemented by optional private health insurance. Diagnostic and treatment facilities include pathology laboratories, haematology laboratories, and public hospitals with facilities located throughout the region, including radiation oncology services and private clinics. There is centralized, organized screening for colorectal, breast, and for cervical cancers. The National Cancer Plan and Programme is implemented at regional and public health levels [4].

In terms of health status, these three territories face a high prevalence of chronic diseases with type 2 diabetes [5], hypertension [6], stroke [7, 8] and end-stage renal disease [9] and infectious diseases [10–12](e.g. Zika, Dengue, HIV). These unfavourable socio-economic and health indicators are associated with a specific epidemiological profile of cancers in these territories.

A previous study on cancer incidence and mortality of solid tumors was performed in mainland France over the period 1980–2012 and showed a decrease in the incidence of breast cancer since 2005. Mortality remained relatively constant until about 1995, and declined thereafter [1].Despite stabilising between 2003 and 2010, incidence of breast cancer has been on the rise again in more recent years, i.e. 2010–2018 (mean + 0.6% per year).Cervical cancer rates have been declining for several decades in mainland France, which can be explained by the introduction of screening programs, Human papilloma virus detection and vaccination [13, 14]. Mortality also declined regularly in mainland France over the same period.

In Guadeloupe, a PBCR study was conducted for the period 2008–2013 and indicated a higher incidence of breast cancer, but with lower rates compared to mainland France [15]. In Martinique, a study of overall survival of cervical cancers for the period 2002–2011 [16] reported overall survival of 55%, and both incidence and mortality rates were higher than in mainland France. In French Guiana, cancer research studies have mainly focused on breast and cervical cancers [3, 17–19]. Although overall, cancer incidence is lower in French Guiana than in mainland France, the incidence of cervical cancer is significantly higher, while the standardized incidence and death rates were lower than in metropolitan France and South America for breast cancer [18].

Our study presents cancer incidence and mortality world-standardized rates in Guadeloupe, French Guiana and Martinique for gynaecological cancers.

## Methods

### Data sources for incidence and mortality

Patient records are reviewed actively. Quality control procedures were designed as recommended by the French Network of cancer registries FRANCIM, in accordance with the International Agency for Research cancer (IARC). Thanks to data cross-matching and control of all available data sources, the registries guarantee high quality cancer registration data.

There are no Death Certificate Only registrations in France; data were extracted from the French epidemiological centre on medical causes of death. In view of the high quality of the database, the data are available through the International Agency for Research on Cancer [20] and through the National French Cancer Institute and Public Health Institute.

### Incidence data

For Guadeloupe, Martinique and French Guiana, incidence data come from population-based cancer registries, and cover the periods 2008–2014, 2007–2014 and 2010–2014 respectively. Incidence data for mainland France were predicted by combining incidence data available in the Departments covered by a registry with healthcare data, using a calibration model [21].

### Mortality data

Mortality data were obtained from the Centre for Epidemiology of the medical causes of death (CepiDC), and cover the period 2007 to 2014. Data are available for all Departments in France. They are coded according to the International Classification of Diseases 10th revision (ICD-10). Due to the fact that a large and variable proportion of death certificates do not distinguish between cervical and endometrial cancer as the cause of death, mortality rates cannot be calculated for each of these two subtypes separately at a regional level.

It should also be noted that mortality data for the year 2012 were not exploitable for Martinique. Therefore, the mortality indicators for 2007–2014 do not include the year 2012 for Martinique.

### Statistical methods

Standardized incidence and mortality rates were calculated using the world population of the World Health Organization (WHO) from 1960 as the standard population [10]. These rates correspond to the incidence and mortality that would be observed in this standard population if it was subjected to the incidence and mortality observed. They are expressed per 100,000 person-years.

The standardized incidence ratio (SIR) or standardized mortality ratio (SMR) in a given geographic area (Department or region) is the ratio of the estimated number of incident cases (or deaths respectively) to the expected number of cases (or deaths), if the incidence (mortality) rates by age group in that geographical area were identical to those of mainland France.

## Results

### All cancers

Over the period 2007–2016, the number of new cancer cases was estimated at 356,109 per year in mainland France, of which 44.7% occurred in women. The world-standardized incidence rates for all cancers combined were 261.1 per 100,000 person-years in women. Standardized mortality rates were 74.3 per 100,000 person-years in women. In the regions of Guadeloupe, Martinique and French Guiana, all cancers combined represented 3567 new cases per year, of which 1420 (39.9%) occurred in women, and 1517 deaths per year of which 659 deaths (43.4%) in women.

Table 1 presents situation in Martinique, Guadeloupe and French Guiana as compared to mainland France, the annual number of new cases and deaths, standardized incidence and mortality rates, standardized incidence and mortality ratios, with 95% confidence intervals.

**Table 1:** Annual number of new cases and deaths for breast, cervical, corpus uteri and ovarian cancers, standardized incidence and mortality rates, standardized incidence and mortality ratios, with 95% confidence intervals

	Incidence						Mortality					
	New cases^1^		WSR^2^		SIR^3^		Deaths		WSR^2^		SMR^3^	
All Women												
Guadeloupe	584	[566; 602]	166.7	[161.2; 172.4]	0.63	[0.61; 0.65]	283	[271; 295]	65.3	[62.3; 68.4]	0.86	[0.83; 0.90]
Martinique	624	[607; 642]	168.4	[163.2; 173.7]	0.66	[0.64; 0.67]	322	[309; 336]	67.5	[64.3; 70.9]	0.92	[0.89; 0.96]
French Guiana	212	[199; 225]	202.9	[190.4; 216.1]	0.79	[0.74; 0.83]	54	[49; 59]	57.7	[52.1; 63.8]	0.76	[0.69; 0.84]
Mainland France	159,093	[157,095; 161,124]	261.1	[257.7; 264.5]			63,416	[63,242; 63,591]	74.3	[74.1; 74.6]		
Breast												
Guadeloupe	215	[204; 226]	65.8	[62.4; 69.4]	0.65	[0.61; 0.68]	49	[45; 54]	13.1	[11.8; 14.7]	0.78	[0.71; 0.86]
Martinique	204	[195; 215]	60.6	[57.5; 63.8]	0.60	[0.57; 0.63]	51	[46; 57]	12.8	[11.4; 14.5]	0.77	[0.69; 0.85]
French Guiana	56	[50; 63]	52.9	[46.7; 59.8]	0.54	[0.48; 0.61]	11	[9; 13]	10.8	[8.5; 13.5]	0.73	[0.58; 0.90]
Mainland France	53,172	[52,420; 53,937]	97.7	[96.3; 99.1]			11,640	[11,566; 11,715]	15.5	[15.4; 15.6]		
Cervix												
Guadeloupe	28	[24; 32]	8.7	[7.4; 10.2]	1.35	[1.16; 1.55]						
Martinique	26	[22; 30]	7.2	[6.1; 8.5]	1.24	[1.07; 1.42]						
French Guiana	25	[20; 29]	22.5	[18.5; 27.1]	3.13	[2.60; 3.74]						
Mainland France	3159	[3020; 3307]	6.6	[6.3; 7.0]								
Corpus uteri												
Guadeloupe	42	[37; 47]	10.7	[9.5; 12.2]	1.05	[0.93; 1.17]						
Martinique	30	[26; 34]	7.8	[6.8; 9.0]	0.72	[0.63; 0.82]						
French Guiana	9	[6; 12]	8.4	[6.0; 11.6]	0.89	[0.65; 1.20]						
Mainland France	6951	[6834; 7070]	10.5	[10.3; 10.6]								
Ovarian												
Guadeloupe	17	[14; 21]	5.7	[4.7; 7.1]	0.62	[0.51; 0.74]	14	[11; 16]	3.5	[2.8; 4.4]	0.72	[0.59; 0.87]
Martinique	14	[11; 16]	4.6	[3.7; 5.8]	0.47	[0.38; 0.56]	14	[12; 18]	3.4	[2.7; 4.4]	0.72	[0.59; 0.87]
French Guiana	9	[6; 12]	8.9	[6.4; 12.2]	1.10	[0.79; 1.48]	4	[3; 6]	4.7	[3.2; 6.8]	1.03	[0.71; 1.45]
Mainland France	4782	[4659; 4908]	7.7	[7.50; 7.93]			3590	[3548; 3631]	4.4	[4.41; 4.53]		

(1) Incidence mainland France: 2007–2016; Guadeloupe: 2008–2014;Martinique: 2007–2014; French Guiana: 2010–2014.(2) World-standardized rates: rates are standardized to the age structure of the world standard population and expressed per 100,000 person-years. (3) Ratios standardized to mainland France

A total of 1583 new cancer cases per year (all sites) were reported in Martinique (39.4% in women). In Guadeloupe and French Guiana, respectively 1528 (38.2% in women) and 456 (46.5% in women) cancer cases were reported.

In women, the most common type is breast cancer (Martinique 33% - Guadeloupe 37% - French Guiana 26%), well ahead of colorectal cancer (Martinique 14% - Guadeloupe 12% - French Guiana 8%). In French Guiana, cervical cancer is the second site (12%). In Martinique, stomach and cervical cancer represent 5% each in third position. In Guadeloupe, corpus uteri is the third site (7%) – (data not shown).

Guadeloupe and Martinique present similar world-standardized incidence rates; they are lower than that observed in women in French Guiana. Regarding mortality, world-standardized mortality rates are also similar between Guadeloupe and Martinique, and higher than in French Guiana.

Overall, the 3 regions have standardized incidence and mortality rates that are lower than the national average in both sexes, with pronounced under-incidence and under-mortality (more than 10% lower than in mainland France (Table 1)), with the exception of mortality in women in Martinique (8% lower).

All details, results, and the full report (in pdf format), are available at: https://www.santepubliquefrance.fr/maladies-et-traumatismes/cancers/articles/estimations-regionales-et-departementales-de-l-incidence-et-de-la-mortalite-par-cancer-en-france-2007-2016

### Breast

Over the period 2007 to 2016, an average of 53,172 women were diagnosed with breast cancer in mainland France each year (Table 1), accounting for 33% of incident cancer cases in women. An average of 11,640 deaths per year was reported in mainland France over the period 2007–2014, corresponding to 18.4% of cancer-related deaths in women.

Breast cancer is the second most common cancer type in the French overseas territories, and the most common type in women. With 215 new cases in Guadeloupe, 205 in Martinique and 56 in French Guiana each year, breast cancer represents respectively 37, 33 and 26% of incident cancer cases in women in these 3 regions. In the French overseas territories, world-standardized incidence rates are 65.8 per 100,000 person-years in Guadeloupe, 60.6 in Martinique and 52.9 in French Guiana. Incidence is 35 to 46% lower than in mainland France, placing the French overseas territories among the regions with the lowest incidence rates in France. Breast cancer is the leading cause of cancer-related death in women in the French overseas territories. Mortality is lower in all 3 regions compared to mainland France, but the standardized mortality ratios are lower than those for incidence, varying from 22 to 27%. Standardized mortality is 13.1 per 100,000 person-years in Guadeloupe, 12.8 in Martinique and 10.8 in French Guiana, which is lower than in mainland France (15.5).

### Cervix

From 2007 to 2016, an average of 3159 women were diagnosed with cervical cancer each year in mainland France (Table 1), representing 2% of incident cancer cases in women.

In the regions of Guadeloupe, Martinique and French Guiana, cervical cancer is diagnosed in an average of 79 women per year (Table 1), i.e. 5.6% of all incident cancers in women. The incidence rate is higher than in mainland France for all regions with a particularly high rate observed in French Guiana compared both to mainland France and the regions of Guadeloupe and Martinique.

### Corpus uteri (endometrial cancer)

Over the period 2007–2016, an average of 6951 women was diagnosed each year in mainland France (Table 1), i.e. 4.4% of incident cancer cases in women.

In the regions of Guadeloupe, Martinique and French Guiana, endometrial cancer is diagnosed in an average of 81 women per year, corresponding to 5.7% of incident cancer cases in women. A higher number of cases are recorded in Guadeloupe, compared to Martinique, yielding an incidence rate of endometrial cancer that is similar in Guadeloupe to mainland France, whereas incidence rates are lower in Martinique and French Guiana than in mainland France.

### Ovarian cancer

Ovarian cancer was diagnosed in an average of 4782 women in mainland France each year for the period 2007–2016, accounting for 3% of incident cancer cases (Table 1).

In the regions of Guadeloupe, Martinique and French Guiana, ovarian cancer is diagnosed in an average of 40 women each year (Table 1), corresponding to 2.8% of incident cancer cases in women.

Guadeloupe and Martinique show comparable rates, and both have lower incidence rates than mainland France. Conversely, French Guiana has a higher, albeit non-significant incidence rate compared to mainland France. In total, 32 deaths were recorded, i.e. 4.9% of cancer-related deaths in women. For all 3 regions, no significant difference in mortality was observed compared to mainland France.

## Discussion

The incidence of all cancers combined is currently lower in the departments of the French overseas territories than in mainland France, but is following a negative trend, likely due to the ageing of the population and the increased prevalence of risk factors linked to lifestyle (sedentary lifestyle, overweight and obesity [22], tobacco smoking [23]). Preventive measures targeting these modifiable risk factors will be key to fighting against many types of cancers.

The incidence of breast cancer is highest in developed countries, particularly in France, which, along with the countries of Northern and Western Europe, has especially high incidence [24]. After a substantial increase up to the year 2005, the incidence of breast cancer declined sharply and then stabilized after 2008 [1, 25]. Despite a reduction observed since the middle of the 1990s, mortality remains high. Breast cancer nonetheless has a good prognosis, with net survival at 5 years of 88% for cancers diagnosed between 2005 and 2010 [26].

Although lower than in mainland France, the incidence of breast cancer in the French overseas territories was on the rise over the period 2008–2014. In Guadeloupe, this is reflected by a lower average age at diagnosis (56 years), with more than a third of cases occurring in women aged less than 50 [15], thus raising the question of the age groups targeted for organized screening.

The mean childbearing age was 30 years in Martinique and Guadeloupe, and 28 years of age in French Guiana between 2005 and 2015. During the latest 5-year period (2010–2015), a mean of 2.19 and 1.98 children per women was observed respectively for Guadeloupe and Martinique. In French Guiana, United Nations statistics report an average of 3.42 children per woman [27]. The main risk factors for breast cancer are related to hormonal and reproductive functions (early puberty, late menopause, older age when having first child, low number of children, no breast-feeding, use of hormone replacement therapy). Other risk factors have also been identified, including alcohol consumption, obesity after menopause, low levels of physical activity, and tobacco smoking [28]. Aging is recognized as the main risk factor for breast cancer, and the increasing age profile in both Martinique and Guadeloupe will cause steep increases in breast cancer occurrence [29]. A systematic review examined the state of the evidence regarding the influence of social determinants of health on breast cancer risk factors in the Caribbean [30]. The authors reported that Caribbean women with indicators of a lower socioeconomic position could be at a higher risk of breast cancer as they reported higher alcohol intake, obesity, and limited breastfeeding.

Genetic predisposition reportedly accounts for 5 to 10% of breast cancers, notably through alterations of the BRCA1 and BRCA2 genes [31]. Improved knowledge of the variants underlying hereditary cancers and improved access to genetic testing will need to be developed in the future in the Caribbean [32].

Furthermore, breast cancer incidence is also impacted by screening practices. The rate of participation in organized screening, which has been implemented across all of France since 2004, was 51% in 2015–2016 for women aged 50 to 74 years, but this rate varies across Departments [33]. Individual screening also exists, but is less well documented.

The incidence of cervical cancer is lower in developed countries that have been implementing screening using the Papanicolau smear test for many years. Together with the countries of Northern and Western Europe, France is among the countries with the lowest incidence of cervical cancer [24]. Incidence and mortality from cervical cancer have been declining steadily since the 1980s, although the decrease has slowed somewhat since the 2000s [1]. Net survival at 5 years for women diagnosed between 2005 and 2010 was 64% [26]. In the French overseas territories however, cervical cancer still has a high incidence rate, particularly in French Guiana.

Cervical cancer is caused by persistent infection within the cervix with high oncogenic risk subtypes of the sexually transmitted human papillomavirus (HPV) [13]. Active smoking, the existence of other genital infections, long-term use of oral contraceptives, and acquired immune deficiency can predispose to the persistence of infection or progression towards cancer. Epidemiological studies have been performed in the French overseas territories into the profile of HPV infections [34–36] and showed that it is necessary to take into account the epidemiological specificities and HPV seroprevalence observed in the French overseas territories. These studies showed epidemiological specificities in HPV genotyping. A study of 540 women with normal cervical cytology living in remote villages of French Guiana showed that 27.2% of women with normal cervical cytology had a positive HPV test. The main HPV genotypes were HPV 53(3.52%), 68(3.33%), 52(2.59%), 31(2.22%) and 16 (1.85%). This study also reported a prevalence of HPV 16 of 6.8% among HPV-infected women [36].

The downward trend in incidence and mortality of cervical cancer is largely explained by individual screening with smear tests since the 1960s. However, screening coverage remains suboptimal in France, and was reported to be 62% in 2010–2012 in Departments covered by an organized screening programme [37]. The National Cancer Plan for 2014–2019 planned to expand organized screening to the whole country in 2018, and set a target participation rate of 80% [38]. Since 2007, primary prevention of cervical cancer is possible thanks to vaccination of adolescents against high risk HPV types. The effects of vaccination on incidence and mortality will only start to appear in the medium term, firstly because of the long latency time between high-risk HPV infection and the appearance of lesions, and secondly, because of the very low vaccine coverage rate currently observed. Cervical cancer could become rare in the future if available primary and secondary prevention measures were optimally implemented.

The incidence of corpus uteri cancer is highest in developed countries. In France, compared to other European countries, the standardized incidence rate is lower than the European average [24]. Since the 1980s, incidence has been stable, and mortality has declined slightly [1]. Prognosis is good overall, with net survival at 5 years of 74% for cases diagnosed between 2005 and 2010 [26].

Endometrial cancer occurs predominantly post-menopause and is most often diagnosed based on clinical signs (metrorrhagia) when still at the localized stage. It occurs primarily as adenocarcinoma of the endometrium. The main risk factors are high endogenous (early menarche, late menopause, nulliparous women) and exogenous oestrogen levels (hormone replacement therapy that is not, or poorly compensated by progesterone, use of tamoxifen) [39]. Metabolic risk factors also exist, notably obesity and diabetes, as well as genetic determinants (Lynch syndrome, family history in a first-degree relative). Conversely, long-term use of combined oral contraceptives, regular physical exercise and tobacco smoking are all associated with a lower risk of endometrial cancer [40, 41]. Trends in the incidence and geographical distribution of endometrial cancer could also be influenced by the prevalence of women who have undergone hysterectomy for benign indications [42].

Due to the fact that a large and variable proportion of death certificates do not distinguish between cervical and endometrial cancer as the cause of death, mortality rates cannot be calculated for each of these two subtypes separately at a regional level.

The incidence of ovarian cancer is higher in developed countries [24]. In France, the standardized incidence rate is similar to the average in Eastern European countries, but lower than the average of other European countries. Incidence and mortality have been declining steadily since the 1980s [1, 43], but ovarian cancer mortality remains high, with 3590 deaths from ovarian cancer recorded each year in mainland France over the period 2007–2014, corresponding to 5.7% of cancer-related deaths in women. Net survival at 5 years was 43% for women diagnosed between 2005 and 2010 [26]. With a very low number of cases each year, no significant differences were found for ovarian cancer for the 3 regions compared to mainland France. Nevertheless, a higher trend was observed for French Guiana for both incidence and mortality.

There are a large number of histological subtypes of ovarian cancer, and each has its own specific epidemiological, etiological and prognostic characteristics. Most often, it occurs in the form of epithelial tumours, predominantly high grade serous carcinoma. Risk factors for these tumours are mainly linked to hormonal and reproductive factors. Factors that contribute to decreasing the number of ovulation cycles during a woman’s life reportedly have a protective effect (late puberty, early menopause, parity, breastfeeding, use of oral contraception). Conversely, early menarche, late menopause and the use of hormone replacement therapy are known risk factors [44]. Several other risk factors have also been studied including tobacco, alcohol, obesity, physical exercise, diet and exposure to asbestos or talc, with results that are sometimes conflicting, or that only show a relationship with one or more histological subtypes [45]. A genetic predisposition is thought to account for 5 to 10% of ovarian cancers, mainly through alterations of the BRCA1 gene, and more rarely, the BRCA2 gene [31]. The significant increase in post-cancer survival is leading many patients to cope with the after-effects of oncology treatments, which incurs a potential risk of impaired fertility. The risk of infertility in women after cancer ranges between 40 and 80% depending on their age, the type of cancer (topology, histology) and the type of treatment [46]. Parental projects and fertility are an essential part of quality of life for patients and their families.

The main limitation of our study is the lack of data on socioeconomic status, which is not recorded in the registry. Socioeconomic inequalities in French overseas territories are more pronounced than in mainland France. Compared to the mainland, there is a lower median income, larger income inequalities, and a higher rate of unemployment in the overseas territories. At the crossroads of poor and highly developed areas, French Guiana shows a disparity in socio-economic living standards and lifestyles, linked to multiethnicity. The population benefits from the national French health insurance system, which guarantees universal access to care to all French citizens and to immigrants living legally in the country, depending on administrative and socio-economic conditions. The disparity observed in socio-economic levels in these territories could contribute to social inequalities in cancer care access.

In a recent study on PBCRs data of Martinique and Guadeloupe, the association between cancer incidence and the socioeconomic level of the residence area was analysed [47]. A specific index of social deprivation from census data at a small area level was created, using Bayesian methods. In this study, there was no clear association between area-based deprivation and the incidence of all cancers combined. Women living in the most deprived areas had a higher incidence of stomach (Relative Risk (RR) 1.77, CI 1.12–2.89), breast (RR 1.15, CI 0.90–1.45), and cervical (RR 1.13, CI 0.63–2.01) cancers and a lower incidence of respiratory cancer (RR 0.65, CI 0.38–1.11, 47]. We found no significant association between deprivation and breast or cervical cancer incidence, with a main limitation due to the small number of cases and the consequent lack of statistical power.

## Conclusion

In this study, we performed a comparative analysis of the incidence and mortality data from the three population-based cancer registries of the Caribbean zone. Similar profiles are observed for Martinique and Guadeloupe, whereas French Guiana presents some different characteristics among the gynaecological cancers. Due to their specificities, these registries contribute to the development of cancer surveillance in this area and may serve as benchmarks for estimating cancer burden. There is a higher incidence of cervical cancer, which is a target for prevention through vaccination. Public health programs must therefore take into account the epidemiology of cancer in order to implement public health actions for populations and professionals. These data will contribute to the development of operational objectives in public health for the fight against cancer, especially for women in the Caribbean.

## Data Availability

All details, results, and the full report (in pdf format), are available at: https://www.santepubliquefrance.fr/maladies-et-traumatismes/cancers/articles/estimations-regionales-et-departementales-de-l-incidence-et-de-la-mortalite-par-cancer-en-france-2007-2016

## Abbreviations

ASCO: American Society of Clinical Oncology
BRCA: BReastCAncer
CepiDC: Centre for Epidemiology of the medical causes of death
HIV: Human immunodeficiency virus
HPV: Human papilloma virus
IARC: International Agency for Research on Cancer
ICD: International Classification of Diseases
RR: Relative Risk
SIR: Standardized incidence ratio
SMR: Standardized mortality ratio
WHO: World Health Organization
